# Hunger Artists: Yeast Adapted to Carbon Limitation Show Trade-Offs under Carbon Sufficiency

**DOI:** 10.1371/journal.pgen.1002202

**Published:** 2011-08-04

**Authors:** Jared W. Wenger, Jeffrey Piotrowski, Saisubramanian Nagarajan, Kami Chiotti, Gavin Sherlock, Frank Rosenzweig

**Affiliations:** 1 Department of Genetics, Stanford University, Stanford, California, United States of America; 2 RIKEN Advance Science Institute, Chemical Genomics Research Group, Wakoshi, Japan; 3 Division of Biological Sciences, The University of Montana, Missoula, Montana, United States of America; Washington University School of Medicine, United States of America

## Abstract

As organisms adaptively evolve to a new environment, selection results in the improvement of certain traits, bringing about an increase in fitness. Trade-offs may result from this process if function in other traits is reduced in alternative environments either by the adaptive mutations themselves or by the accumulation of neutral mutations elsewhere in the genome. Though the cost of adaptation has long been a fundamental premise in evolutionary biology, the existence of and molecular basis for trade-offs in alternative environments are not well-established. Here, we show that yeast evolved under aerobic glucose limitation show surprisingly few trade-offs when cultured in other carbon-limited environments, under either aerobic or anaerobic conditions. However, while adaptive clones consistently outperform their common ancestor under carbon limiting conditions, in some cases they perform less well than their ancestor in aerobic, carbon-rich environments, indicating that trade-offs can appear when resources are non-limiting. To more deeply understand how adaptation to one condition affects performance in others, we determined steady-state transcript abundance of adaptive clones grown under diverse conditions and performed whole-genome sequencing to identify mutations that distinguish them from one another and from their common ancestor. We identified mutations in genes involved in glucose sensing, signaling, and transport, which, when considered in the context of the expression data, help explain their adaptation to carbon poor environments. However, different sets of mutations in each independently evolved clone indicate that multiple mutational paths lead to the adaptive phenotype. We conclude that yeasts that evolve high fitness under one resource-limiting condition also become more fit under other resource-limiting conditions, but may pay a fitness cost when those same resources are abundant.

## Author Summary

Microorganisms such as yeast have been used for decades to study adaptive evolution by natural selection. Thirty years ago in now seminal experiments, a strain of yeast was evolved multiple times under carbon limitation. The adaptive changes that gave rise to increases in fitness have previously been studied both phenomenologically and mechanistically but not in detail at the molecular level. To better understand the basis for these strains' fitness increase, we sequenced their genomes and identified putative adaptive mutations. We found that multiple mutational paths lead to these fitness increases. We also determined whether the evolved yeasts' gains in fitness under the original conditions in some cases diminished fitness under other conditions. We therefore evaluated their performance relative to the ancestral strain under the evolutionary and two alternative resource-limiting conditions by determining the ancestral and evolved strains' relative fitnesses and gene-expression levels under all three conditions. We found scant evidence among evolved strains for fitness trade-offs when nutrients were scarce, but discovered a cost was paid when nutrients were plentiful.

## Introduction

R.A. Fisher's fundamental theorem of natural selection relates the rate of adaptation by populations of organisms to their genetic variance in fitness at a given time [1]. Understanding the mechanistic basis for this variance, and the distribution of a population's fitness variance under alternative modes of selection, have been goals of evolutionary biologists since the modern synthesis. Experimental laboratory evolution using metazoans such as *Drosophila* [2], and microorganisms such as bacteria [3], algae [4], or yeast [5] has provided the most direct route to these goals, providing deep insight into the forces that guide the adaptive process under different modes of selection [6].

In foundational work, Paquin and Adams monitored the evolution of laboratory strains of the budding yeast *Saccharomyces cerevisiae* [7] during growth under aerobic glucose limitation in continuous culture [8], [9]. By monitoring population genetic dynamics over the course of these experiments and characterizing the fitness phenotypes of individual evolved clones, they arrived at two key insights concerning the mechanism of adaptive evolution in clonal populations. First, adaptive shifts, inferred from scoring fluctuations in the frequency of neutral markers, occurred more often in evolving diploids than in otherwise isogenic haploids [8]. Second, in a subset of evolutions, relative fitness of successive adaptive clones was non-transitive, that is, although any particular clone was more fit than its immediate predecessor it was not necessarily fitter than the ancestral strain used to found the population [9]. As for the specific mechanisms underlying changes in fitness, common phenotypes among adaptive clones included increased glucose transport capacity and characteristic cell morphology changes that increased surface area to volume ratios, as might be expected for cells adapted to better scavenging low concentrations of limiting growth substrate [10].

The clones derived from Paquin and Adams' original experimental evolutions have shown an enduring usefulness over the past 25 years for addressing fundamental questions concerning the nature of adaptive evolution. 15 years after the original experiments, Brown *et al.*, discovered that at least one genetic mechanism underlying enhanced glucose transport was tandem duplication of adjacent genes encoding the high-affinity glucose transporters Hxt6 and Hxt7 [11]; this genomic rearrangement has subsequently been observed in other independent glucose-limited evolution experiments [12]. Ferea *et al.* [13] probed more deeply into physiological changes that result from prolonged glucose-limited selection using one of these strains and two others evolved under identical conditions from the same ancestor. In the first experiment to use gene-expression microarrays in evolutionary biology [3], they showed an overall shift in these clones from fermentation to respiration, in what they termed an “enhanced classical Pasteur effect” that allowed for more efficient metabolism of the available glucose [13]; subsequently, Dunham *et al.* used these same clones to discover genomic rearrangements that occur during adaptation [14].

One fundamental question that these strains have not yet been used to address is: “Does evolution of increased fitness under one type of selection cause decreased fitness under another?” - in other words, are there fitness trade-offs? That trade-offs occur and constrain organismal evolution is foundational to much evolutionary theory, theory that extends into ecology where it has guided analyses of how communities are structured in relation to resource availability [15]–[17] and which factors constrain life history evolution [2], [18]. The question of how niche breadth evolves has been addressed both theoretically and experimentally [19]–[22]. It is widely held that adaptation to a homogenous environment should favor a narrowing of niche-breadth, whereas adaptation to a heterogeneous environment should favor evolution of a broad niche and maintenance of population genetic variation [19], [23]. One theory for why niche breadth might be narrowed in an environment where selection is uniform and constant is based on the possibilities that either adaptive mutations or neutral mutations that accumulate under one selection pressure are deleterious under others – possibilities known, respectively, as antagonistic pleiotropy or mutation accumulation. However, these trade-offs can be hard to demonstrate directly and mechanistically [24]–[26], in part because they must be tested in relation to the ancestral state, which may not always be known or accessible.

Correlated responses of fitness to selection are conventionally measured in terms of how well an organism performs in an environment different from the one in which it evolved [24], [27], [28]. However, the observation of correlated responses does not by itself prove the existence of trade-offs. What is required are experimental data showing that in alternate environment(s) fitness is reduced relative to the ancestor [29]. Experimental microbial evolution has shown that trade-offs do occur, but not inevitably, following selection. Clones from populations of *E. coli* serially diluted for 20,000 generations in minimal medium containing glucose as the sole carbon source exhibit reduced fitness on a variety of alternative carbon sources [30]–[32]. Narrowed niche-breadth does not appear to be specific to evolution on a particular nutrient, as populations of *E. coli* experimentally adapted to low temperature can show trade-offs at high temperature [33]–[35]. Experimental evolution using the facultatively photosynthetic algae *Chlamydomonas* has also revealed trade-offs: strains evolved in the presence of light often grow more poorly than the original ancestor in the dark, and vice versa [27], [29]. In a final example, among *E. coli* populations evolved in a continuous, mixed-sugar chemostat environment (lactulose and methyl-galactoside), clones evolve most often through either amplification of the *lac* operon or mutations in the *mgl* operon. In only one out of thirteen chemostats did a clone evolve having mutations in both of these operons [36]. Taken together these experimental studies suggest that fitness trade-offs (which could be due to either antagonistic pleiotropy [AP] or mutation accumulation [MA]), while not inevitable, can play an important role in determining an organism's niche breadth. In addition to these studies, other recent work has delved deeply into the molecular genetic basis for adaptation using gene-expression analysis, targeted gene sequencing, array comparative genomic hybridization [aCGH], and/or whole genome sequencing [12], [37]–[41]. Notwithstanding such breakthroughs, few attempts have been made to comprehensively integrate whole-genome sequence data with estimates of physiological performance and fitness; this activity is essential to achieving the goals of understanding the mechanistic basis for population genetic variance, as well as for the distribution of fitness variance under alternative modes of selection.

Here we present just such an integrated set of fitness, physiological, and whole-genome sequence data that we use to test whether evolutionary adaptation to one type of carbon limitation diminishes organismal performance under other types of carbon limitation (or non-limitation). Specifically, we asked whether the well-studied Paquin and Adams [7]–[9] and Ferea *et al.* [13] yeasts that were evolutionarily adapted to aerobic glucose limitation fared better, no differently, or worse than their common ancestor when cultured in two other carbon-limited environments: anaerobic glucose limitation in chemostats or aerobic acetate limitation in chemostats. Additionally, we assayed these strains' fitness under non-limiting glucose in serial batch culture, and in glucose-rich, nitrogen-limited chemostats. Remarkably, we discovered that evolved strains were consistently more fit than their common ancestor under every condition where carbon was limiting, but that this advantage disappeared when carbon was abundant, indicating the existence of a trade-off. To understand how this might be so, we measured for each strain in each environment indicators of physiological performance including yield and global gene-expression profiles. Then, to discover the genetic mechanisms that underlie these phenotypes and to further unravel the evolutionary history of these well-studied clones, we sequenced the genomes of all five adaptive clones and their common ancestor.

## Results

### Evolved Clones Outperform Their Ancestor in Diverse Carbon-Limited Environments

Paquin & Adams [8], [9] and Ferea *et al.* [13] isolated end-clones from independent evolution experiments originating from a diploid strain of S288c (CP1AB) that was grown under continuous aerobic glucose limitation [7]. To determine whether five of these clones from independent lineages (hereafter referred to as E1 through E5, see Materials and Methods) maintained their fitness advantage relative to the ancestor in “novel” carbon-source environments, selection coefficients were calculated by competing each clone and their ancestor against a common reference strain (see Materials and Methods) in three environments: aerobic glucose limitation (the “direct” fitness response, i.e., to the original selection), anaerobic glucose limitation and aerobic acetate limitation (the latter two measure “correlated” responses). The two alternative carbon-limiting environments were chosen to test their effects on the “enhanced classical Pasteur effect” observed in these clones by Ferea *et al.* [13]. Specifically, they provide complementary environments to test the fitness consequences of adaptively switching from respiro-fermentative metabolism to respiration alone. In one case, only fermentation is possible (anaerobic glucose limitation), while in the other, only respiration is possible (aerobic acetate limitation). Competitions were carried out for approximately 20 generations, which was short enough to ensure no further adaptive genetic changes would appreciably affect the outcome.

**Figure 1:**
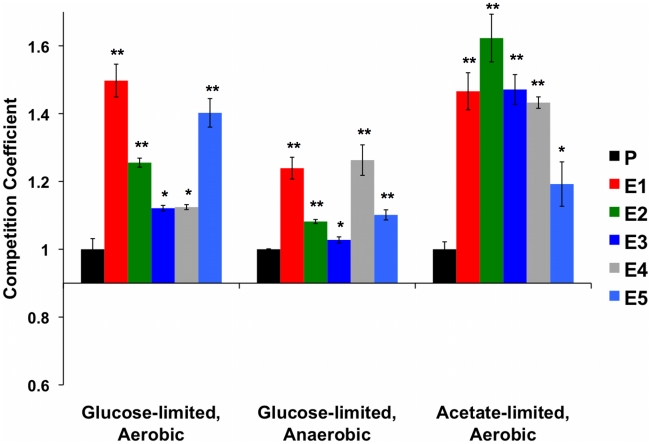
Normalized Competition Coefficients for 3 Environments. Data are competition coefficients calculated by competing each strain (evolved and ancestor CP1AB) against a common reference strain. Values are the average of three biological replicates, with values normalized such that ancestral CP1AB (“P”) equals 1 in each environment. Significant differences versus CP1AB within each environment were calculated using a 2-tailed t-test. “*” indicates p<0.05 and “**” p<0.01. Error bars represent the Standard Error of the Mean. See Table S1 for un-normalized, average competition coefficients.

As expected, each evolved clone had a significantly higher relative fitness than the ancestor in the aerobic glucose-limited environment in which all of the original evolutions were performed (Figure 1 (“Aerobic”) and Table S1). In the alternative carbon-limited environments, i.e., “Anaerobic” and “Acetate”, each adaptive clone also exhibited significantly higher fitness relative to the ancestral diploid CP1AB, (in a 2-tailed t-test) (Figure 1 and Table S1). These data indicate that the adaptation to aerobic glucose limitation in each of the clones is not accompanied by a reduction in fitness compared to the ancestral state in either of two alternative environments. They also suggest that selection has improved these clones' ability to scavenge the limiting nutrient and has also enhanced respiratory efficiency.

In the above analyses, we compared evolved clones' direct and correlated responses to selection within each environment. To compare the responses to selection between environments, we calculated grand means of all five clones' relative fitnesses within each environment and tested these means for significance differences between each of the three environments (2-tailed t-test). We hypothesized that some of the adaptive mutations due to the original aerobic regime would be deleterious or neutral under anaerobic growth, specifically those that resulted in the “enhanced classical Pasteur effect.” This hypothesis predicts that these same mutations would produce a fitness advantage in aerobic acetate-limited growth (provided they continue to enhance respirative growth). The data in Table S2 show significant differences in overall mean fitness between all three environments. As predicted by our hypothesis, mean relative fitness in the aerobic glucose-limited environment is higher than in anaerobic glucose limitation (competition coefficients 0.280 vs. 0.143 respectively, *p*<0.05 in two-tailed t-test); strikingly, the relative fitness under acetate-limitation is higher still than under aerobic glucose limitation (competition coefficients 0.437 vs. 0.280 respectively, *p*<0.05 in two-tailed t-test). These data are consistent with the notion that while the mutations conferring the adaptive advantages for E1 through E5 have an overall net positive effect on fitness when compared to the original ancestor in each environment, some of these mutations might be deleterious or neutral in the anaerobic environment. More specifically, these results indicate that increased glucose transport is still advantageous under anaerobic glucose limitation, but that enhanced respiration provides no benefit in the absence of oxygen. However, enhanced respiration provides obvious benefits under acetate limitation, where oxygen is available.

While these trends are important, they are not universally true for all clones. For example, one clone (E4, Figure 1) has the highest relative fitness in the anaerobic environment, yet it has one of the weakest fitness advantages compared to the other four clones in aerobic glucose limitation and an intermediate fitness under acetate limitation. There are also two cases (E1 and E5) in which the correlated responses in the acetate-limited environment are less than the direct fitness response under aerobic glucose limitation. Taken together, these two observations are consistent with a hypothesis that the more adapted to one environment a particular clone might be, the higher the chance that there will be a fitness cost in a different environment, and vice-versa. This variation in fitness also suggests that multiple genetic paths that have answered the selection are represented among these independent clones.

### Physiological Responses Help Explain Correlated Fitness Advantage

**Figure 2:**
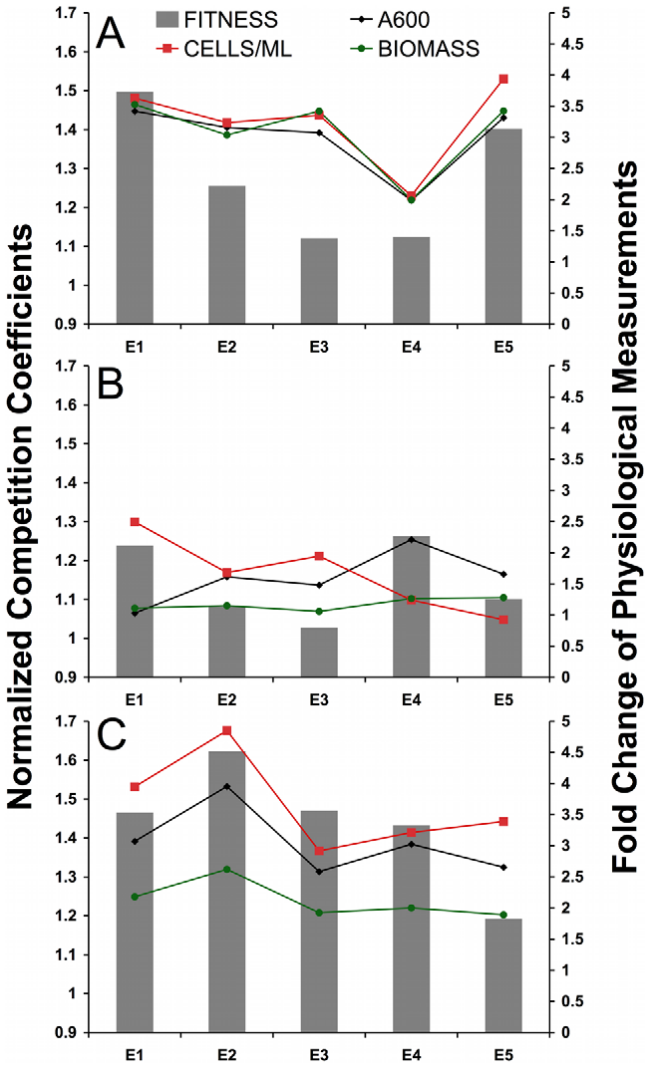
Physiological Fold-Changes Overlaid on Fitness. Representation of physiological data combined with fitness data for three environments A) aerobic glucose limitation, B) anaerobic glucose limitation, C) aerobic acetate limitation. Primary (left) y-axis is normalized competition coefficient (same normalization as Figure 1). Secondary (right) y-axis is fold change (evolved/ancestral) of steady state physiological data including A600 (optical density at 600 nm), cells mL^−1^, and biomass g 100 ml^−1^. See Table S3 for un-normalized values and statistical analysis.

To determine whether unique physiological traits are associated with the fitnesses we observed, three independent, single colonies of each evolved clone and the common ancestor were grown to steady state in the chemostat and three different parameters of culture growth—culture density (optical density [OD] at 600 nm), cell number (cells mL^−1^), and biomass (g 100 mL^−1^)—were measured (Table S3, Figure 2). The data in Figure 2 are represented as fold-change relative to the ancestor; please see Table S3 for raw values and statistics. For the aerobic glucose-limitation growth condition, we observed, for all five evolved clones, the same physiological changes reported in previous work [10], [11], [13], namely a two to four-fold increase with respect to the ancestor in optical density, cell number, and biomass (Table S3, Figure 2A). The observed increases in all measured cell growth parameters among the evolved clones have been postulated to arise from both an increase in glucose transport and from an adaptive switch to increased rates of respiration, resulting in a more energetically efficient use of the available glucose [13].

In alternative carbon-limited environments, evolved clones also demonstrate increased cell yield, relative to their common ancestor, although these differences are much more pronounced under aerobic acetate limitation than under anaerobic glucose limitation. In fact, the differences in magnitude we observe under acetate limitation are comparable to those we observed under aerobic glucose limitation, with values ranging from an almost 5-fold increase in one case (cells mL^−1^ for E2), down to a roughly 2-fold increase in biomass for E5 (Figure 2C, Table S3). In the case of anaerobic glucose limitation, however, the increases are more modest, ranging from a maximum 2.5-fold increase (cells mL^−1^ for E1 over CP1AB) to no change or perhaps even a slight reduction (cells mL^−1^ for E5 and OD600 for E1) for these traits (Figure 2B, Table S3). Another general observation is that under both acetate limitation and aerobic glucose limitation, there seems to be general concordance between the three physiological traits measured, i.e., changes relative to the ancestor in biomass, cell number, and culture density change in the same direction and with similar magnitudes when considering any individual clone. This is to be contrasted with anaerobic glucose limitation, where there is no concordance between any of the three physiological parameters; in fact, the data are consistent with steady state OD and cells mL^−1^ being anti-correlated. Finally, we observe a clear relationship between relative fitness increases and the magnitude of the growth parameter increases relative to the ancestor for these three physiological parameters, both in the aerobic glucose-limited environment (Figure 2A) and to a lesser extent in the acetate-limited environment (Figure 2C). However, none of the three parameters seem to be correlated to relative fitness in the anaerobic environment, suggesting that mechanisms independent of enhanced respiration are contributing to higher relative fitness under anaerobiosis in these clones compared to the ancestor (Figure 2B).

### Direct Gene-Expression Responses Are Not Constitutive in Alternative Carbon-Limited Environments

The “enhanced classical Pasteur effect” described by Ferea *et al.* [13] was inferred from their gene-expression microarray data. We, too, have used microarrays to determine how the transcriptome responds to alternative carbon-limited environments when cell populations are at steady state. RNA was isolated from the same cultures that were used to estimate physiological parameters (using two of the three biological replicates), and transcript abundance was measured on Agilent yeast catalog arrays, relative to a pooled reference that contained equimolar amounts of each sample. The values (Log_2_(sample/reference)) for the biological replicates were averaged for the subsequent analyses.

**Figure 3:**
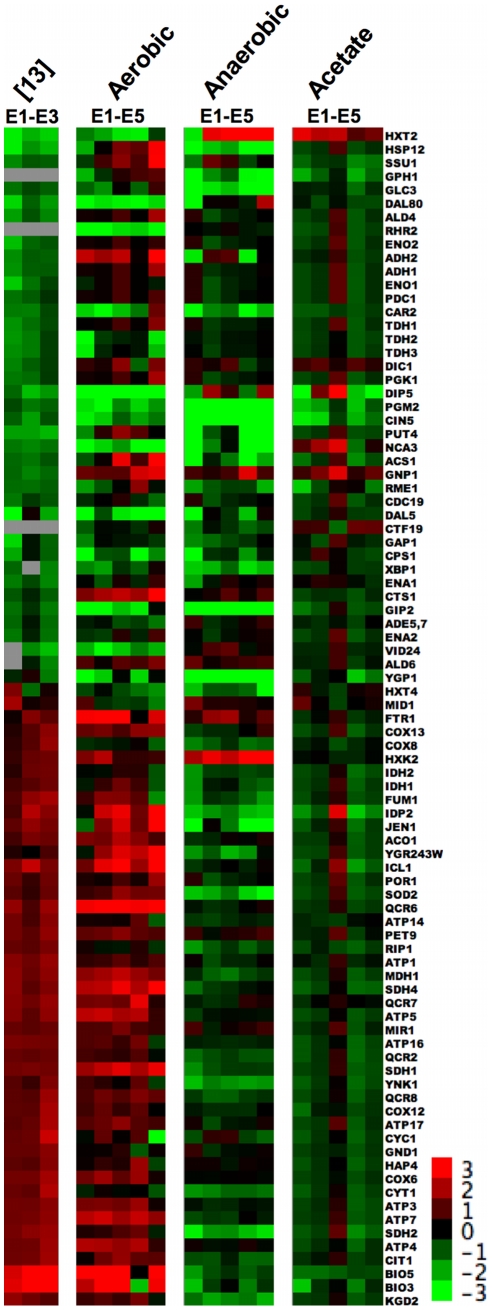
88 Genes that Show “Enhanced Pasteur Effect” from Ferea *et al.* Average of two biological replicates of relative mRNA abundance (measured against a pooled reference of all samples) for all three environmental conditions of the 88 genes identified in Ferea *et al.* [13] whose expression level in that experiment was >2 fold up or down-regulated relative to the ancestor. All data are normalized to the ancestor (evolved log2(sample/reference) – ancestral log2(sample/reference)). Gene tree has been removed for space considerations. Clustered raw data are available in Dataset S1.

Because Ferea et al. [13] performed gene expression microarray analysis with only three of these evolved clones (E1 through E3) we wished to determine if the “enhanced classical Pasteur effect” also occurred in the other two clones (E4 and E5) under aerobic glucose limitation, as well as whether the effect manifested in the two alternative environments. We therefore examined our microarray data alongside the 88 “enhanced classical Pasteur effect” genes shown in Figure 1 of Ferea *et al.* (Figure 3, Dataset S1).

Under aerobic glucose limitation our data (Figure 3) largely recapitulate the “enhanced classical Pasteur effect” described by Ferea *et al.* [13], as well as evolved transcriptional changes reported under aerobic glucose limitation by Jansen, et al. [42]. In all adaptive clones the expression levels of genes involved in glucose oxidation increased while levels of glycolytic genes decreased, relative to the common ancestor. Note that the previously uncharacterized E4 and E5 clones appear to share many of the changes observed in E1, E2, and E3 in the aerobic glucose-limited environment, although not exclusively; in fact, E5 appears to be the most divergent of the five evolved clones. Some deviations from the original experiments are seen, particularly in a number of the glycolytic genes that do not appear as highly repressed in our experiments as in the original work (*ADH1*, *ADH2*, *ENO1*, *ENO2*, *PGK1*, *PDC1*).

Under anaerobic glucose-limiting conditions, glucose-oxidation pathways are repressed (or simply not induced) as would be expected in the absence of oxygen. Contrary to our initial expectations, these same pathways are not highly expressed relative to the ancestor under aerobic acetate limitation. Thus, at least for this set of genes, it appears that evolved clones do not have mutations in one or more global regulators of pathways that result in constitutive up-regulation. Interestingly, under aerobic and anaerobic glucose limitation (but not acetate limitation) we observed up-regulation of the hexokinase gene *HXK2*. *HXK2* encodes a bifunctional enzyme whose cytosolic form irreversibly commits glucose to metabolism by phosphorylating it [43]; also, the nuclear form of Hxk2 is required for Mig1-dependent glucose repression of multiple genes, including *HXK1* and itself [44]. Although this enzyme is thought to be a key element of the high-glucose sensing pathway [45], in chemostats fed with 0.08% glucose *HXK2* expression was increased 4- to 16-fold in all five evolved clones compared to the parent, a result we provisionally attribute to a 2-fold decrease in expression of the Mig1-Hxk2 regulator *SNF1* observed under this condition.

### Evolved Clones Share Gene-Expression Responses Consistent with Up-Regulation of Nutrient Signaling Pathways

**Figure 4:**
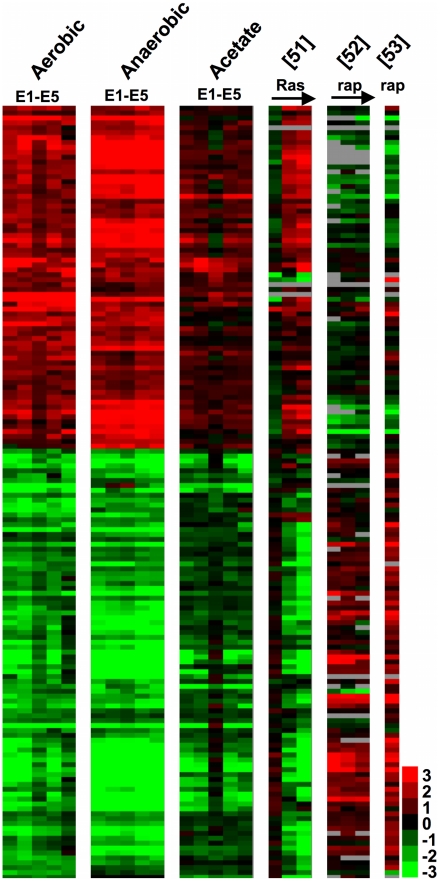
Expression Changes Common to Evolved Clones. Significance analysis was performed as a 2-class SAM between all evolved clone data and all ancestral data for the three conditions. Prior to clustering the data were normalized to the ancestor as for Figure 3. Data from [51] represents a time course of a constitutive *Ras2^G19V^* allele induced by the *GAL10* promoter. mRNA abundances at 0, 20, 40, and 60 min were measured on an Affymetrix platform and normalized for our purposes to 0 min (log2(20 min/0 min), etc.). Data from [52] are relative mRNA abundances over a time course (0, 15, 30, 60 min) of rapamycin (rap) treatment normalized to 0 min (log2(15 min/0 min), etc.). Data from [53] are relative mRNA abundance of rapamycin treated wild type cells versus wild type (log2(rapamycin/no treatment)). Each row represents a gene, and grey indicates missing data. Clustered raw data are available in Dataset S2.

To uncover general expression patterns underlying the direct and correlated responses to selection in these strains, we performed a two-class, unpaired Significance Analysis of Microarrays [SAM] [46], comparing all of the data for the evolved strains in all three environments to all of the data for the ancestor. This procedure identified 160 genes whose expression values significantly differed between the evolved and ancestral strains (FDR<5%) (Figure 4, Dataset S2). Because the Ras and TOR pathways provide obvious candidates for a general adaptive response that could lead to improved growth of evolved clones relative to their ancestor in all three environments [12], [47]–[50], we also considered how these 160 genes behaved in three publicly-available datasets: one that assayed gene-expression in response to induction of *RAS1* [51]; and two that measured gene-expression when cells were treated with rapamycin, a drug that inhibits the TOR pathway [52], [53]. What is visually striking about this list of genes is the degree of correlation (or anti-correlation in the case of TOR) with the up-regulation of *RAS1*, and the down regulation of the TOR pathway. GO::TermFinder [54] analysis supports this visual observation, as the up-regulated genes are enriched for functions including ribosome biogenesis (GOID 42254, Bonferroni corrected p-value = 4.67e-5), while down-regulated genes are enriched for response to oxidative stress (GOID 6979, Bonferroni corrected p-value = 3.3e-6) and [small molecule/vacuolar/protein] catabolic process (GOID 9056, Bonferroni corrected p-value = 1.71e-06) among others. These three functions all have regulatory ties to both TOR and Ras/cAMP signaling [55]–[57] and support the hypothesis that mutations that modulate signaling through the Ras/cAMP and/or TOR pathways are likely to provide a mechanism for the evolution of a broad niche that encompasses multiple carbon-limited environments.

### Whole-Genome Sequence Analysis of E1–E5 and CP1AB Reveals a Surprising Number of Mutations That Are Likely Adaptive under Nutrient Limitation

The genomes of CP1AB and the five evolved clones E1–E5 have been previously interrogated in a number of different ways including southern blot [11], gene-expression microarrays [13], array comparative genomic hybridization [14], and most recently by whole-genome tiling arrays [39]. While some of the genomic events that have occurred as a result of adaptation are known – notably, *HXT6/7* amplifications [11], [14], rearrangements near the *CIT1* locus [14], and mutations in the *AEP3* gene [39] – a comprehensive resequencing of these strains has not been performed. We therefore performed high-throughput whole-genome sequencing (Table S4) and then determined single-nucleotide polymorphisms [SNPs], small insertions and deletions [indels], and larger-scale genomic copy number variations [CNVs] in each evolved strain relative to the common ancestor (see Materials and Methods). We validated each substitution or indel in both the evolved and the ancestral strains by Sanger sequencing of the locus, using the same genomic DNA sample that was used for whole-genome sequencing, isolated from a single colony of the indicated strain (for primers, see Table S5). Table 1, Table 2, Table 3, Table 4, and Table 5 show the results of this analysis, which identified 28 single-nucleotide polymorphisms relative to the ancestor in E1 (evolved for 460 generations), 17 in E2 (250 generations), 11 in E3 (250 generations), 9 in E4 (301 generations), and 10 in E5 (264 generations), as well as two short indels, one each in E1 and E2. One general observation is that the strain that underwent the most number of generations of selection (E1) contained the most polymorphisms relative to the other evolved strains. Another general observation is that these strains have accumulated polymorphisms at a faster rate than haploid populations evolved under almost identical conditions; a haploid adaptive clone isolated from one of the populations from Kao & Sherlock [12] after 440 generations had only accumulated 5 SNPs, 1 transposon insertion, and the *HXT6/7* amplification [58]. This observation supports Paquin and Adams' original conclusion that diploids accumulate adaptive mutations more rapidly than haploids [8].

**Table 1:** Summary of Substitutions and Indels for E1 (460 generations).

Chr	Pos	Ref	Alt	Zygosity	Annotation	Syn?	Gene
II	40357	A	T	hom (Ref)->het	CDS	I 405 L	*BNA4*
II	336311	C	G	hom (Ref)->het	CDS	A 170 P	*REB1*
II	649740	G	A	hom (Ref)->het	CDS	V 619 I	*NGR1*
IV	63483	C	A	hom (Ref)->het	CDS	Q 512 H	*CDC13*
IV	1154148	T	C	hom (Ref)->het	intergenic		*HXT7*
IV	1154160	A	G	hom (Ref)->het	intergenic		*HXT7*
V	246512	A	T	hom (Ref)->het	intergenic		*SAP1/CAJ1*
V	246513	G	A	hom (Ref)->het	intergenic		*SAP1/CAJ1*
VII	96460	A	G	hom (Ref)->het	CDS	syn	*MIG2*
VII	674719	A	C	hom (Ref)->het	CDS	K 845 T	*VAS1*
VIII	71474	G	T	hom (Ref)->het	CDS	G 400 V	*YHL017W*
VIII	142574	C	G	hom (Ref)->het	CDS	E 329 Q	*DED81*
X	514538	G	T	hom (Ref)->hom (Alt)	CDS	V 162 F	*NUP85*
XI	248086	G	A	hom (Ref)->het	CDS	syn	*YKL102C*
XI	365899	A	C	hom (Ref)->het	CDS	syn	*RGT1*
XII	353829	A	T	hom (Ref)->het	CDS	S 3304 T	*MDN1*
XII	445330	C	A	hom (Ref)->het	intergenic		*YLR152C/ACS2*
XII	855305	C	T	hom (Ref)->het	CDS	S 36 L	*YLR365W*
XIII	136753	C	A	hom (Ref)->het	CDS	S 418 R	*POB3*
XIV	399951	C	A	hom (Ref)->het	CDS	G 196 V	*TOM70*
XIV	429367	A	C	hom (Ref)->het	CDS	K 545 Q	*MET4*
XIV	619219		+A	hom (Ref)->het	CDS	−169aa	*SIS1*
XV	172862	T	G	hom (Ref)->het	CDS	L 598 W	*IRA2*
XV	780679	T	G	hom (Ref)->hom (Alt)	intergenic		*SNR17A/DFR1*
XVI	113951	G	A	hom (Ref)->het	CDS	G 1767 D	*FAS2*
XVI	422593	C	G	hom (Ref)->het	CDS	S 549 *	*MUK1*
XVI	489680	C	A	hom (Ref)->het	CDS	R 562 L	*SVL3*
XVI	549443	C	T	hom (Ref)->het	CDS	P 320 S	*AEP3*
XVI	640448	A	C	hom (Ref)->het	CDS	Q 308 H	*ARP7*

In the “Syn?” column, amino acid changes are indicated if the mutation is non-synonymous. Stop codons are indicated by “*”. For intergenic mutations, flanking or nearby genes are indicated in the “Gene” column.

**Table 2:** Summary of Substitutions and Indels for E2 (250 generations).

Chr	Pos	Ref	Alt	Zygosity	Annotation	Syn?	Gene
II	589713	T	G	hom (Ref)->het	intergenic		*FZO1/DTR1*
III	52717	G	T	hom (Ref)->het	CDS	G 25 C	*GID7*
III	303345	A	G	hom (Ref)->het	intergenic		*YCR101/2*
IV	573016	G	T	hom (Ref)->het	CDS	V 790 F	*MAK21*
IV	677840	G	T	het->hom (Ref)	intergenic		*FOB1/ALT2*
VII	120132	G	T	hom (Ref)->het	CDS	T 259 K	*MCM6*
VII	845690	G	C	hom (Ref)->het	tRNA		*tG(GCC)G1*
VIII	85164	G	T	hom (Ref)->het	intergenic		*YAP3/tRNA-Val*
VIII	335011	G	T	hom (Ref)->het	CDS	T 218 N	*YHR112C*
VIII	490972		-G	hom (Ref)->het	CDS	−202aa	*NVJ1*
X	427840	G	T	hom (Ref)->het	CDS	C 895 F	*CYR1*
XII	898188	G	C	hom (Ref)->het	5′ UTR		*RPS29A*
XIII	687242	C	A	hom (Ref)->het	CDS	V 15 L	*YMR209C*
XV	183953	C	A	hom (Ref)->het	CDS	L 758 I	*AVO1*
XV	466475	G	T	hom (Ref)->het	CDS	V 569 L	*SGO1*
XVI	549443	C	A	hom (Ref)->het	CDS	P 320 T	*AEP3*
XVI	549453	A	T	hom (Ref)->het	CDS	E 323 V	*AEP3*
XVI	549454	A	G	hom (Ref)->het	CDS	syn	*AEP3*

**Table 3:** Summary of Substitutions and Indels for E3 (250 generations).

Chr	Pos	Ref	Alt	Zygosity	Annotation	Syn?	Gene
IV	475252	T	G	hom (Ref)->het	CDS	Y 403 D	*RAD61*
IV	1178957	G	T	hom (Ref)->het	CDS	V 98 L	*SBE2*
VI	200817	C	A	hom (Ref)->het	CDS	T 315 K	*PES4*
VI	215628	C	G	hom (Ref)->het	CDS	P 773 A	*MET10*
VIII	114175	A	G	hom (Ref)->het	CDS	L 248 P	*GPA1*
VIII	405592	G	T	hom (Ref)->het	CDS	C 876 F	*RTT107*
IX	301064	C	T	hom (Ref)->het	CDS	C 65 Y	*YIL029C*
XIII	46350	C	A	hom (Ref)->het	intergenic		*CTK3/BUL2*
XIV	155354	G	T	hom (Ref)->het	CDS	L 85 F	*ORC5*
XV	76249	C	A	hom (Ref)->het	CDS	T 617 K	*ALR1*
XVI	549444	C	A	hom (Ref)->het	CDS	P 320 Q	*AEP3*

**Table 4:** Summary of Substitutions and Indels for E4 (301 generations).

Chr	Pos	Ref	Alt	Zygosity	Annotation	Syn?	Gene
II	706287	C	G	hom (Ref)->het	CDS	M 169 I	*ALG7*
VII	147966	G	A	hom (Ref)->het	intergenic		*CDC55/RPS26A*
VII	187253	G	C	hom (Ref)->het	CDS	V 399 L	*SUA5*
VII	332623	T	C	hom (Ref)->het	CDS	K 615 E	*PAN2*
IX	69914	G	C	hom (Ref)->hom (Alt)	CDS	N 1180 K	*SLN1*
XIII	729971	C	A	hom (Ref)->het	CDS	G 385 C	*RRP5*
XV	379626	T	C	hom (Ref)->het	intergenic		*HST3/BUB3*
XV	821295	A	G	hom (Ref)->het	CDS	syn	*PNT1*
XV	878199	T	C	hom (Ref)->hom (Alt)	intergenic		*MBF1/BUD7*

**Table 5:** Summary of Substitutions and Indels for E5 (264 generations).

Chr	Pos	Ref	Alt	Zygosity	Annotation	Syn?	Gene
VII	34321	A	T	hom (Ref)->het	CDS	syn	*ZIP2*
VII	126868	T	C	hom (Ref)->het	CDS	F 724 S	*MDS3*
VII	155424	G	T	hom (Ref)->het	CDS	P 197 T	*STR3*
VIII	389336	A	G	hom (Ref)->het	LTR		*YHRCdelta10*
IX	166517	C	A	hom (Ref)->het	intron		*MOB1*
X	566107	C	A	hom (Ref)->het	CDS	T 2231 K	*TOR1*
XI	644490	C	G	hom (Ref)->het	intergenic		*SIR1/FLO10*
XII	933374	T	C	hom (Ref)->hom (Alt)	CDS	syn	*YLR407W*
XV	237964	C	A	hom (Ref)->het	CDS	P 1009 Q	*GAL11*
XV	452940	T	A	hom (Ref)->het	CDS	I 175 F	*ALG8*

Based on the fact that these independent diploid yeast colonies that were sequenced were single adaptive clones that each represent one lineage throughout the entirety of the evolution, we used simple coalescent theory to estimate the number of mutations we would expect by chance. Theory predicts that the number of neutral mutations we would expect to see in any given clone is simply μ * L * t where μ = mutation rate (per base per generation), L = genome size (bases), and t = time (generations). Using even the most generous estimate of mutation rate (6.44e-10 per bp per generation estimated by Lang & Murray [59] at the *CAN1* locus) – we would only expect to see a small number of mutations per strain. For E1, we only expect 7–8 neutral mutations, and for E2–E5 we only expect 3–5 mutations. Under the assumption that this is a Poisson process, seeing the observed number of mutations is significant for each clone (p<0.01 for E1, E2, E3, and E4, and p<0.05 for E4, Table S6). These data support the hypothesis that a significant fraction of the mutations that we identified are adaptive. Finally, the vast majority (63 out of 69) of these polymorphisms are heterozygous, as might be expected in evolving diploid populations.

To gain further insights about the nature of these mutations as a group, we characterized them with respect to whether they lie in coding regions and if so, whether missense or nonsense amino-acid substitutions are created. Again under the assumption that the mutational events are distributed across the genome in a Poisson fashion, we can determine whether both the distribution of mutations in coding regions, and the frequency with which mutations within a coding region result in an amino acid change, deviate from our expectations. The probability of a mutation occurring in a coding region (including stop codons) is ∼0.721 and, using clone E1 as an example, the expected number of coding mutations out of 28 observed mutations is between 20 and 21. Given these estimates, our null hypothesis under a Poisson distribution is that we will not observe greater than 20–21 coding mutations out of 28 total mutations. Because 21 of these 28 mutations actually occur in coding regions, we cannot reject the null hypothesis, and thus we do not see more mutations in coding regions than we would expect by chance (p = .085) (Table S7). Similarly for clone E1, we know that 18 of our 21 coding mutations result in an amino acid change. Using the known probability of a coding sequence mutation effecting an amino acid change (see Materials and Methods), our expectation is that ∼0.787 (or 16–17 out of 21) coding mutations will be non-synonymous. Under a Poisson distribution, we again cannot reject the null hypothesis that the number of non-synonymous mutations observed in E1 (18/21) is greater than the expected number (16–17/21) with p = 0.088 (Table S7). These data combined with our previous observation suggest that despite a large fraction of these mutations probably being adaptive, the gene-dense nature of the genome (∼72% coding) and a large probability that a mutation occurring in a coding region will result in an amino acid change (∼79%) does not allow us to predict that any given mutation being non-synonymous means it will necessarily be adaptive.

### Mutational Changes Have Occurred in Glucose Sensing and Transport Pathways As Well As in Mitochondrial Structural Proteins

In light of these data, but with the caveat that segregating out dozens of mutations and individually testing their fitness effects is a large undertaking, we can still draw some interesting conclusions about the biological implications of these mutations from the actual genes that are affected. We observed that the gene *AEP3*, which encodes a mitochondrial integral membrane protein that stabilizes mRNA of the ATP synthase complex [60], is affected by polymorphisms in three of the evolved strains – E1, E2, and E3. This strongly argues for these mutations being adaptive, particularly because each of the mutations creates a different amino acid substitution. These mutations had previously been observed in E2 and E3 [39], and each created a novel growth phenotype on acetate at 37°C in haploid progeny of E2 and E3 [14]. These mutations, now also confirmed in E1, presumably confer an adaptive phenotype during growth in limiting glucose and in limiting acetate despite being heterozygous. A number of other genes that have been mutated are clearly connected to adaptation in the original evolution condition, notably those involved in glucose transport and its regulation (*MIG2*, *RGT1*), as well as glucose and nutrient signaling (*IRA2*, *CYR1*, *AVO1*, *TOR1*, *ARP7*). In particular, we discovered mutations in the Ras/cAMP signaling pathway that have been previously identified in glucose-limited evolutions [12], strongly suggesting adaptive roles for *IRA2*, one of the Ras-GTPase-activating proteins [61], and *CYR1*, which encodes the yeast adenylate cyclase [62]. Signaling through the TOR pathway has also been implicated in physiological adaptation to limiting glucose [63], again suggesting adaptive phenotypes for mutations in *TOR1* and *AVO1*, a member of the TORC2 complex [64]. These mutations in the Ras/cAMP and TOR pathways have clear implications for the changes that we observed in the transcriptomes of these evolved clones. In E1 we also observe a mutation in *MUK1*, which has no known function, but which has also been the target of selection in independently-evolved, haploid, glucose-limited populations of yeast; the mutation appears not to be adaptive on its own ([12], [58]) suggesting the presence of epistasis between mutations in these strains. Taken together, our data reveal likely genetic bases for adaptation to glucose limitation in diploid yeasts, including changes in pathways affecting glucose/nutrient signaling, regulation of glucose transport, and enhancement of aerobic respiration, as well as other intriguing mutations whose roles in adaptation remain to be elucidated.

To confirm the DNA copy number changes and other larger scale genome rearrangements discovered by Dunham, *et al.* [14], we applied a depth-of-sequence-coverage approach [37] to identify areas of increased or decreased coverage relative to the ancestor, CP1AB (see Materials and Methods). Figure S1 shows the genome mean-centered log2 ratio of coverage (evolved/ancestor) in E1 through E5. Our data recapitulate those of [14], specifically the ChrIV (*HXT6/7*) amplification and ChrXIV rearrangement in E1 (Figure S1, E1 and E1, *HXT6/7*), the ChrVII amplification and ChrXV deletion in E4 (Figure S1, E4), and the ChrIV amplification and ChrXIV deletion in E5 (Figure S1, E5). While the biological significance of all of these structural variants remains to be elucidated, the specific rearrangements in both E1 and E5 near the important TCA cycle gene *CIT1* on ChrXIV, as well as the specific amplification of the *HXT6/7* chimera in E1 and of the right arm of ChrIV in E5 (which includes the *HXT6* and *HXT7* loci) have clear implications for adaptation to carbon-limited growth.

### Where Are the Trade-Offs? An Example of Antagonistic Pleiotropy under Glucose Non-Limiting Conditions

**Figure 5:**
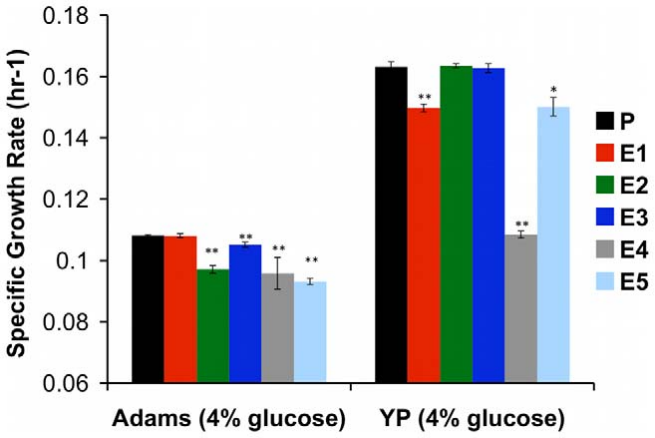
Specific Growth Rate of Evolved Clones Is Decreased in Glucose-Rich Environment. Maximum specific growth rates of evolved clones and ancestor CP1AB (“P”) were calculated by growing multiple independent colonies of each strain in batch culture in two different media (rich “YP” medium and minimal “Adams” medium) with 4% glucose. Values are the mean of 3 independent colonies (change in ln(OD) per hour during exponential growth), with error bars showing standard error of the mean. Significant differences versus CP1AB within each medium were calculated using a 2-tailed t-test. “*” indicates p<0.05 and “**” p<0.01.

Glucose at high concentrations is toxic to cells [65], and glycolytic intermediates can produce reactive carbonyl species that damage DNA and proteins [66]. Not surprisingly, yeast tightly regulates glucose flux into glycolysis by coordinating expression of low-, medium- and high-affinity hexose transport genes in response to changing concentrations of extracellular glucose [67]. Given these observations and our own observations of the increased copy number of *HXT6/7* and mutations in *RGT1* and *MIG2* discovered by genome sequencing, an obvious candidate condition in which to test for the presence of trade-offs is glucose-rich medium, as enhanced glucose transport may no longer be advantageous when glucose is abundant, and may even be costly. To test this hypothesis, we grew evolved and ancestral clones under glucose non-limiting conditions in batch culture and found that nearly all showed diminished maximum specific growth rate (μ_max_), relative to their common ancestor (Figure 5).

**Figure 6:**
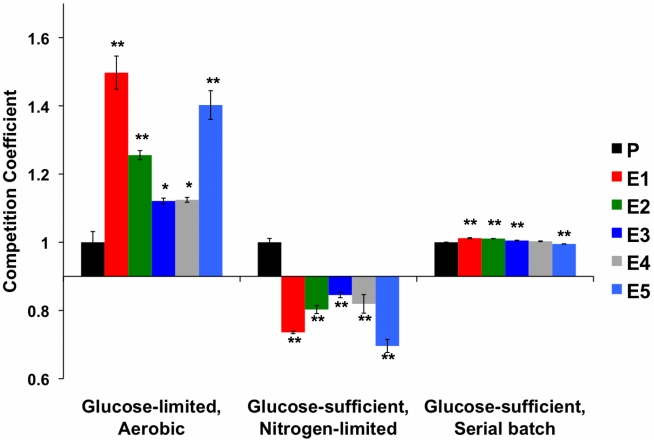
Normalized Competition Coefficients for 2 Glucose-Rich Environments. Data are competition coefficients calculated by competing each strain (evolved and ancestor CP1AB) against a common reference strain. Values are the average of three biological replicates, with values normalized such that ancestral CP1AB (“P”) equals 1 in each environment. Significant differences versus CP1AB within each environment were calculated using a 2-tailed t-test. “*” indicates p<0.05 and “**” p<0.01. Error bars represent the Standard Error of the Mean. See Table S8 for un-normalized, average competition coefficients. Aerobic Glucose-limited data are the same as in Figure 1 and were added for comparison.

To determine whether evolved strains' diminished growth rate on glucose translated into fitness differences when this resource was abundant we competed the strains and their common ancestor against the same reference strain as before, under two continuous conditions: nitrogen-limited, glucose-sufficient chemostat, and glucose-sufficient serial batch cultures (Figure 6 and Table S8). Under both conditions, the fitness advantages observed under carbon limitation disappeared. In serial dilution, evolved strains performed no, or only very slightly, better than their ancestor (while statistically significant, the effect sizes are only ∼1%), and in NH_4_+ limited, carbon-sufficient chemostats, evolved strains were invariably out-competed by their ancestor. Thus, these evolved yeasts are specifically adapted to growth on carbon as a limiting resource, and these adaptations are either of no benefit or actually detrimental when that resource is abundant.

Because amplification of high-affinity hexose transporters appears to be in negative epistasis with adaptive mutations in *MTH1* [58], we further tested for the possibility that high *HXT6/7* copy number is disadvantageous under glucose-sufficient conditions. We founded 5 independent populations with CP1AB and 10 independent populations with the E1 clone (which contains this amplification in addition to other putative adaptive mutations), experimentally evolved these for >100 generations by serial transfer in 2% YEP dextrose medium and then tested for changes in *HXT6/7* copy number by quantitative PCR. We discovered that in at least one instance, copy number decreased (Figure S2), indicating that this condition can favor reduction or loss of the amplification. Longer-term experiments in rich media will be required to determine whether lower-copy number variants are consistently selected. To determine whether the *HXT6/7* amplification alone decreases the growth rate relative to a strain with wild-type *HXT6/7* copy number, we characterized spores derived from a diploid strain that was heterozygous for this amplification and carried no other adaptive mutations (spores courtesy D. Kvitek). We observed that the *HXT6/7* amplification resulted in decreased growth rate relative to sister spores containing the “wild-type” *HXT6/7* locus (Figure S3). These data support the hypothesis that the *HXT6/7* amplification is deleterious during growth in excess glucose, and hence is an example of antagonistic pleiotropy.

## Discussion

This work builds upon seminal experiments in evolutionary biology performed nearly 30 years ago by Paquin and Adams. The original experiments shed light on the topography of fitness landscapes in evolving asexual populations and the tempo of adaptive change in relation to ploidy [8], [9]. Later experiments using these yeasts yielded the first global-scale insights into how evolution shapes the transcriptome [13] and brings about chromosomal rearrangements via recombination at rRNA loci, Ty- and deltaelements [14]. We have used these strains to ask questions concerning direct and correlated responses to selection, the evolution of niche breadth, and the complete catalog of mutations that accumulate within diploid yeasts independently derived from a single common ancestor. The answers to these questions make it possible to begin to elucidate the molecular mechanisms underlying observations made in each of the previous foundational studies.

### Adaptive Clones Independently Evolved under One Type of Carbon Limitation and Exhibit Similar Phenotypes under Others

Our choice of assay regimes was motivated by a desire to understand the generality, relative magnitude and mechanistic bases of adaptations for acquiring [11] and processing [13] limiting glucose under prolonged selection. All evolved strains showed significant improvement in fitness under the selective regime and assay regimes where carbon was limiting. Microarray analysis of cells grown under the selective regime showed that, relative to their ancestor, evolved yeasts had diminished expression of genes in fermentative metabolism and increased expression of genes in oxidative metabolism. These results essentially recapitulate earlier findings [13], even though our analyses were performed on a different platform and included strains from the Paquin and Adams experiments that had not been previously investigated. Interestingly, beginning with a different yeast ancestor (CEN.PK 113-7D) and using a slower dilution rate (0.1 h^−1^), Jansen, et al. [42] also saw diminished fermentative capacity in yeast evolved under prolonged glucose limitation, evidenced at both the transcriptional and enzymatic levels. Thus, adaptive evolution of an “enhanced classical Pasteur effect” under this selective regime appears to be a general result.

Remarkably, although the clones we investigated evolved in independent populations under aerobic glucose limitation, they performed better than their common ancestor in other carbon limiting environments under both anaerobic and aerobic conditions. The adaptive clones' superior performance is manifest in cell yield and fitness in both the selective and assay regimes. The relative magnitudes of the physiological values associated with fitness differences can be easily explained in terms of the energetics of aerobic vs. anaerobic catabolism [68] and the phenotypes most likely to bring about a competitive advantage under resource limitation: either or both enhanced capacity to scavenge limiting resource which increases fitness without significant gains in yield, or increased efficiency of limiting resource utilization resulting in higher cell yield and higher fitness [17], [69]. Specifically, we suggest that the heritable changes we identified that improve glucose uptake capacity in aerobic conditions [10] result in the modest increased yield and fitness under anaerobic glucose limitation; under aerobic acetate limitation, we see heritable changes that improve aerobic capacity, evidenced by more pronounced changes in these parameters.

The relative magnitude of yield and fitness differences in these two assay regimes reflects the scope for selection: only modest gains are possible in high-affinity glucose transport, whereas much more substantial gains are possible by shifting to and then improving upon the machinery of oxidative metabolism, whose ATP yield is many-fold greater than fermentation [68]. Thus, cells have greater scope for adaptive change by simply enhancing the classical Pasteur effect. That said, a striking result of our data is that adaptation to glucose limitation has not only resulted, as expected, in increased glucose transport and diminished catabolite repression, but also to more efficient machinery for carrying out oxidation, even of the non-fermentable, non-repressing substrate, acetate.

### Convergent Adaptive Phenotypes Arise from Different Sets of Mutations Affecting Common Pathways

Our phenotypic data indicate that five independently evolved clones have converged on growth phenotypes that give them a competitive advantage in selective (and carbon scarce) assay regimes alike. We would not necessarily have predicted this from previous studies [11], [13], [14], as they provided few clues as to the possible costs of adaptive change. Remarkably, the similar phenotypes we observed arise from different sets of mutations in each clone, although certain genes and pathways seem more likely to be targeted by selection than others. For example, while increased glucose transport is clearly an adaptive phenotype, it appears to have been accomplished by different mutations in two of these clones. Clone E1 contains mutations in two genes that regulate glucose transporter gene-expression, *MIG2* and *RGT1*, as well as a tandem duplication of the genes encoding the hexose transporters Hxt6 and Hxt7. Interestingly, the mutations in *MIG2* and *RGT1* are synonymous, indicating potential functional roles for mutations that do not effect an amino-acid change. By contrast, clone E5 contains an amplification of the entire right arm of chromosome IV, containing the *HXT6* and *HXT7* loci.

Our microarray results, viewed through the lens of our whole genome sequencing data, suggest other adaptive mechanisms additional to the changes in *HXT6/HXT7* copy number and *CIT1* regulation noted previously [11], [14]. These gene-expression data in particular lend support to the hypothesis that the glucose/nutrient signaling pathways of these strains are affected in such a way as to promote cell division even in a nutrient-poor environment. Two clones have mutations that likely affect signaling through the Ras/cAMP pathway. E1 is heterozygous for a mutation in *IRA2*, a gene that encodes a Ras-GAP that functions to decrease intracellular cAMP levels. Interestingly, E2 contains a mutation in the same pathway in the gene that encodes yeast adenylate cyclase itself, *CYR1*. These mutations would be particularly interesting to characterize, as our gene expression data and data from [12] would predict that the *cyr1* mutation would be a gain-of-function mutation that increases intracellular cAMP levels, whereas the *ira2* mutation should be a loss-of-function mutation resulting in constitutive Ras signaling and similar increases in cAMP levels.

Gene-expression data also suggest increased signaling through the TOR pathway across all evolved clones, relative to their common ancestor, an observation that is again supported by the presence of novel mutations in this pathway. We found that E2 has a mutation in *AVO1*, a component of the TORC2 complex, while E5 has mutations in *TOR1* itself as well as in *MDS3*, a putative component of the TOR signaling cascade. Again, our prediction is that these mutations should be gain-of-function that increase signaling through this key regulatory pathway.

Finally, our fitness and physiological data point to increased function in oxidative metabolism as an alternate mode to answer the challenge of limiting glucose, while simultaneously creating a fitness advantage when grown in acetate limitation. We uncovered three independent mutations in *AEP3*, a gene that encodes a mitochondrial protein important for ATP synthase function. Strikingly, the mutations in E1, E2, and E3 all affect the same codon but effect independent amino acid substitutions, and E2 contains 2 additional nucleotide changes that change another amino acid in close proximity to the other mutated codon. We observe additional mutations in E1 that likely affect mitochondrial function, including *BNA4* (involved in biosynthesis of nicotinic acid), *NGR1* (over-expression of which impairs mitochondrial function), and *TOM70* (which is a translocase of the outer mitochondrial membrane). It will be illuminating to follow-up these observations with a characterization of their individual or epistatic fitness contributions. These data also provide a hypothesis for observed variation in correlated responses to selection. The whole genome sequence of clone E3 provides no mechanistic basis for enhanced glucose transport, but does have a mutation in the integral mitochondrial protein, *AEP3*. Significantly, we observed that relative to the ancestor and to other independently evolved clones, E3 exhibited highest fitness under acetate limitation and less of a selective advantage under glucose limitation. A final observation regarding clones E3 is that it appears to have the most distinct gene expression pattern compared to the other five clones under acetate limitation (Figure 3 and Figure 4), possibly suggesting roles for altered transcription leading to higher relative fitness under acetate-limited growth. One possible contributor to the observed gene expression differences of E3 under acetate limitation is the mutation in *GPA1*, an upstream G-protein that leads to activation of a transcription factor, Ste12p, that plays a role in both pheromone response and regulation of invasive growth. Indeed, many of the genes that show altered transcription in Figure 3 and Figure 4 are known Ste12p targets, but again, more work will be required to determine if the mutation in *GPA1* is responsible for the observed gene expression phenotype.

Our sequence data provide a rich resource to begin answering other fundamental questions about the nature of yeast's evolutionary adaptation to a limiting resource: What are the fitness and biochemical effects of each new mutation? Which mutations are adaptive, and which are neutral or mildly deleterious and merely hitchhiking? How pervasive is epistasis between new mutations? And, because most novel alleles are heterozygous, which, if any, are over-dominant? Finally, because we have seen haploids adapt to limiting glucose by similar mechanisms, albeit more slowly, (see genotypes in Kao & Sherlock [12]), we may ask: are mutational differences seen in diploids due to ploidy or due to our sampling not having comprehensively obtained all possible genotypes that can respond to this selection?

This work has also addressed unanswered questions posed by Ferea *et al.* [13] concerning the genetic basis of the “enhanced classical Pasteur effect.” While the specific causal mutations of these gene-expression changes remain to be determined, our data lead us to two conclusions. The first is that these changes are not constitutive: mutations that cause increased expression of glucose-oxidation pathways specifically under aerobic glucose limitation can still be repressed in the absence of oxygen, when their expression is inappropriate. The second is that there appear to be multiple adaptive paths to the same phenotype, in opposition to one of the original hypotheses that there are few [13]. Additional experiments will be required to isolate individual mutations and determine how each, alone and in combination with others, impacts differential regulation of glycolysis and the TCA cycle under the selective and assay regimes.

### Apparent Absence of Trade-Offs under Carbon Limitation Makes Possible the Evolution of “Hunger Artists”

Our work brings new evidence to bear on the longstanding question of how trade-offs influence adaptive evolution. Constant, homogenous environments are widely believed to favor evolution of narrow niches in contrast with heterogeneous environments, which are believed to favor evolution of broad niches [19], [70], [71]. Corollary to this belief is that narrow niches arise from trade-offs due to antagonistic pleiotropy, and/or differences in the rates at which beneficial and/or deleterious mutations accumulate in these different selection regimes [28], [72]. Here we find that clones evolved under constant glucose limitation are, as expected, more fit than their common ancestor in the selective regime, but also more fit in two assay regimes: anaerobic glucose limitation and aerobic acetate limitation.

In retrospect, given the changes we have discovered in strains' physiology, gene expression and genome sequence, the apparent lack of trade-offs under the assay regimes we chose is perhaps not so surprising. An increased capacity to scavenge glucose should produce a fitness advantage in any environment in which glucose is meager; thus the direct genetic evidence we see for this in at least two strains, E1 and E5 (*HXT6/7*), likely outweighs any cost of uselessly increasing glucose-oxidation ability under anaerobic glucose-limitation. Similarly, although increased glucose transport is unlikely to be adaptive under acetate limitation, any cost imposed thereby is likely offset by an increased capacity to oxidize carbon. More generally, we can use a term defined by Bell & Reboud [29] to describe selection in aerobic glucose limitation as *synclinal* – meaning that the direct and correlated fitness responses to this selective regime were positive with respect to the ancestor in all five evolved clones. These conclusions, however, might only apply to carbon (or even particular kinds of carbon) limitation, and in further work it would be appropriate to test these evolved clones under a much more diverse set of environments to determine the breadth of their niche. These types of experiments will be crucial to discerning whether trade-offs exist under other assay regimes and, if so, how mutation accumulation and antagonistic pleiotropy combine to produce them.

### Side-Effects of Adaptation: Do Scavengers Suffer in Times of Plenty?

The Paquin and Adams [7]–[9] and Ferea et al. [13] yeasts evolved under limiting glucose have been used over the last quarter century to address fundamental questions relating to the dynamics and mechanisms of adaptive evolution. Our work continues in that vein, providing evidence to support the conclusion that evolution under one resource limiting condition leads to generalists that are more fit than their ancestor under other resource limiting conditions, but less fit when the original limiting resource is abundant. Additionally, we have sequenced these strains' genomes and provided a list of genetic changes that arose in independent evolution experiments, creating a rich resource of information that can be used to continue studying the mechanisms by which organisms adapt to resource scarcity, as well as the apparent cost to being a “hunger artist” when resources are plentiful. Intriguing as these apparent trade-offs that we have identified may be, more work will be required to understand every mutation's mechanistic role (biochemical, metabolic, regulatory, etc.) in adaptation to prolonged resource limitation. We will then be in a position to generate further specific hypotheses as to which conditions should reveal the cost of particular adaptations and whether that cost is incurred as a result of antagonistic pleiotropy, mutation accumulation, or both.

## Materials and Methods

### Strains

Strains used in this study were *Saccharomyces cerevisiae* CP1AB with genotype *MATa/α*, *gal2/gal2*, *mel/mel*, *mal/mal* [7] and evolved clones E1, E4, and E5 [8] and E2 and E3 [13]. The common reference strain used for the competition experiments was DBY11249 (FY4, with a d-Tomato/*NatMX* cassette replacing the dubious ORF *YLR255c*, strain courtesy David Gresham and Greg Lang). Cultures were stored in 15% glycerol at −80°C.

### Competition Experiments

Strains for chemostat cell cultures were grown in 1% YEP Dextrose and 1 mL aliquots were frozen in 15% glycerol at −80°C. The entire contents of a single 1 mL frozen aliquot of either the reference strain and evolved or ancestral isolates were used to inoculate an individual chemostat (ATR SixFors fermentation apparatus, ATR Biotechnologies) with working volume set to 400 mL of minimal (SC) media defined by [73]. Batch cultures were then grown for 24 hours to achieve saturation. After saturation was achieved, chemostat pumps were turned on to the desired dilution rate and 2–3 vessel volumes of turnover were allowed so cultures could reach steady state. 100 mL of each strain growing at steady state (reference plus evolved or ancestor) were transferred to a fresh chemostat and the dilution rate was set to 0.17 hr^−1^ for aerobic (0.08% glucose) and anaerobic glucose limitation (0.08% glucose+420 mg mL^−1^ Tween 80+10 mg L^−1^ ergosterol) and 0.05 hr^−1^ for aerobic acetate limitation (10.9 g/L sodium acetate). For aerobic ammonium limited (0.015% (NH_4_)_2_SO_4_), glucose-sufficient (9 g/L glucose) growth, chemostat dilution rate was also set to 0.17 hr^−1^. Aerobic conditions were achieved by sparging with 25 L h^−1^ of sterile air and anaerobic conditions by sparging cultures with 25 L hr^−1^ sterile-filtered, humidified N_2_ (g). 3 mL samples were taken at time = 0 (immediately following transfer to fresh chemostat) and every 6–8 hours for 2–3 days (∼15 generations). Time and volume of effluent were measured at each sample to determine generations. 1 mL of cells were resuspended in Phosphate Buffered Saline, sonicated for 10 s, and analyzed with flow cytometry to determine relative proportions of fluorescent (reference) to non-fluorescent (experimental sample) strains. 50,000 cells were counted to obtain accurate measurements of relative proportions. Regression analysis of generation time vs. ln(experimental sample/common reference) was used to calculate per-generation competition coefficients. This method is based on the method worked out by Alex Ward and David Gresham and similar to the method used in [74]. Similar procedures were used to compute selection coefficients of strains competed in serial dilution batch culture. The media employed in these experiments was that of Adams et al. with the addition of 4% dextrose (wt/vol). Approximately equal numbers of the test and fluorescent reference strains were combined at an initial cell density of ∼10^5^ cells mL^−1^ in 10 mL media. Samples were cultured for 24 h (∼6.5 generations) at 30°C on a New Brunswick T-7 roller drum, then diluted to a similar cell density in fresh media and cultured an additional 24 h. Samples for FACS analysis were taken over 3 successive serial dilutions (approximately 20 generations). Pairwise competition experiments were performed in triplicate.

To determine specific growth rates in glucose non-limiting batch growth for evolved clones E1–E5, ancestral clone CP1AB, and haploid segregants with or without the *HXT6/7* amplification (haploid segregants GSY2707-2714 were otherwise isogenic from parent diploid GSY1208 that was heterozygous only for the *HXT6/7* amplification), multiple independent single colonies of these strains were grown overnight in 2% YEP dextrose and diluted 1∶50 into fresh medium in a 100 µL, 96-well optical plate (Costar), sealed with optical sealing tape (E&K Scientific), and grown for approximately 24 hours in a TECAN plate reader at 30°C. Specific growth rate was defined as the change in ln(optical density) per hour during exponential growth.

### Experimental Evolution under Nutrient Non-Limiting Conditions

To test the stability of the adaptively evolved *HXT7/6* amplification under nutrient-rich conditions, we selected at random five colonies of the parent strain and ten colonies of adapted strain E1 and used these to found fifteen experimental populations. Populations were inoculated at a density of ∼10^5^ cells mL^−1^ in 10 mL YEPD (2% glucose), cultured at 30°C in a New Brunswick T-7 roller drum, and serially propagated by diluting cells ∼100-fold on a daily basis in fresh media. Experiments were carried out for 15 days (>100 generations); 1 mL of each culture was archived every 25 generations as −80°C 15% glycerol stocks. Population samples from the last time-point of each experiment were spread onto YPD agar. Genomic DNA was prepared from three randomly chosen colonies on each plate using the YeaStar Genomic DNA Kit (Zymo Research); this material was used as template for quantitative PCR assay of *HXT7/6* copy number using primers specific for the *HXT6/7* locus and control primers on chromosome IV designed against the *UBP1* locus (primers in Table S5), using the ΔΔCt method as described by [58].

### Physiological Measurements

To obtain physiological measurements, cultures were grown to steady state in individual chemostats in each of the three environments described above, under identical conditions. Experiments were performed in triplicate. Biomass estimates were determined by rapidly withdrawing 100 mL from fermentation vessels, and fast-filtering this volume through sterile, tared 47 mm, 0.45 mm Nylon filters (Whatman). Filters were dried overnight in an 80°C oven and weighed the following day. Cell number was estimated by haemocytometry using an aliquot from 1 mL of sample treated with 10 ug mL^−1^ cycloheximide. Steady state optical density was measured spectrophotometrically at 600 nm. To isolate total RNA, 100 mL of sample was quickly filtered through 0.45 mm Nylon filters (Whatman) and flash frozen in liquid nitrogen. RNA for gene-expression measurements was isolated using the hot acid-phenol method described by [13].

### Gene-Expression Measurements

To assay relative mRNA abundance, total RNA was isolated as described above. A pooled reference sample was created containing equimolar amounts of each of 36 samples (6 strains in 3 environments, using two of the three biological replicates). 325 ng of total RNA from samples or reference pool was used as the input for reverse transcription and labeling with Cy dyes (Amersham) using the Low RNA-input Linear Amplification Kit (Agilent) following manufacturer's instructions except that reaction volumes were halved. 1.5 µg each of labeled sample and labeled reference were hybridized to Yeast Gene Expression Arrays v2 8×15k (Agilent) for 17 hours at 65°C rotating at 10 rpm in a hybridization oven (Shel Lab). Arrays were then washed according to manufacturer's instructions and scanned at 5 µm resolution on an Agilent Scanner. Data were extracted using Agilent Feature Extraction v9.5.3.1, which uses linear-Lowess normalization and calculates log_2_ ratios. Following data extraction from the raw images, we averaged the data for both probes for each gene. Raw gene-expression data have been deposited in GEO with accession number GSE25081.

### Whole-Genome Sequencing

CP1AB and E1–E5 were streaked for single colonies from 15% glycerol stock solutions (−80°C) onto 2% YEP Dextrose plates. Single colonies were grown in 2% YEP Dextrose liquid cultures at 30°C and genomic DNA was extracted by spooling as described [75]. Paired-end libraries were created using the Illumina Genomic DNA Sample Prep Kit according to manufacturers instructions (5 µg input genomic DNA), and sequencing flow cells were prepared using the Illumina Standard Cluster Generation Kit. Samples were sequenced on the Illumina Genome Analyzer II, and image analysis and data extraction were performed using Illumina RTA 1.5.35.0. Reads were mapped and variants were called using two different methods, with largely similar results. In the first method, reads with qualities (FASTQ) were aligned to the S288c reference genome (SGD, as of Feb 2, 2010) using BWA v0.5.7 [76]. Whole-genome pileup files were generated using SAMtools v0.1.7 [77] and SNPs and Indels were filtered using custom Perl scripts. Briefly, SNPs passed the filter if they were represented in at least 30% of reads in the evolved strain (allowing for heterozygosity) and at most 10% in the ancestor, or at least 80% in the evolved strain but less than 80% in the ancestor (allowing for heterozygous to homozygous mutations). Additional heuristic filters included a confirming read from both strands, with at least 5 reads covering the position in both strains, and no more than one ambiguous SNP call (“N”) or deletion (“*”) at that position. Indels were filtered by requiring at least a 30% or greater allele frequency difference between ancestral and evolved strains, if they shared the same indel call. Additionally, if there were >2 indel calls at a given position, the number of reads supporting the two most common indel calls had to be > = 80% of the total reads covering that position. Raw coverage in evolved and ancestral strains at the given position must also have been at least 10×. In the second method, we mapped reads with qualities using Stampy [78], and applied the Genome Analysis Toolkit (GATK) “Best Practice Variant Detection” [79] by first performing base quality score recalibration, indel realignment, and duplicate removal. We then performed SNP and indel discovery across all evolved and ancestral sequences simultaneously using standard hard filtering parameters [80], and then used custom perl scripts to identify SNP or indel variant calls that were different between ancestral and evolved strains. Primers used to confirm or reject SNPs and Indels are in Table S5. For determining copy number variation (Figure S1), a coverage-based approach was used as outlined by [37]. Briefly, raw sequencing coverage was averaged over 1 Kb intervals across the genome of each evolved clone and the ancestor. Log2(evolved/ancestor) ratios were then calculated and normalized to the genome mean log2 ratio. Genome segments were identified using a circular binary segmentation algorithm implemented in the R software package *DNAcopy* [81] with parameters as follows: data.type [logratio]; smooth.region [3]; alpha sign. cutoff [.01]; min.width [5]; undo.splits [sdundo]; sdundo [4]; nperm [10000]. Raw sequence data have been deposited in the Sequence Read Archive (SRA) database with accession number SRA025083.1.

### Estimate of Probability of Non-Synonymous Mutations

To determine the average probability of a mutation in a coding region effecting a non-synonymous coding change we wrote a custom perl script that calculates the average probability that a mutation would change the codon to encode a different amino acid. Briefly, for each codon in the genome, every possible mutation was generated (9 changes for each codon), and the fraction of those 9 possible mutations that created a non-synonmous codon was recorded. For example, a four-fold degenerate site at the wobble base of a given codon would yield a probability of 2/3 non-synonymous (6 out of 9 mutations change the codon). We then simply averaged this probability across all codons in the genome.

## Supporting Information

Dataset S1 Gene-expression data from Figure 3.
(TXT)

Dataset S2 Gene-expression data from Figure 4.
(TXT)

Figure S1 Evolved Copy Number Variations. Depth-of-coverage plots for E1 through E5, relative to the ancestral diploid CP1AB. Values plotted are log2 ratios of mean sequencing coverage in 1 kb windows across the genome (evolved/ancestral). Red lines represent segment means determined by *DNAcopy* (see Materials and Methods).
(TIF)

Figure S2 Copy Number of *HXT6/7* locus following serial batch evolution under high glucose. Copy number of the *HXT6/7* locus relative to the ancestral parent. Raw values for *HXT6/7* locus were normalized to an internal control chrIV locus (*UBP1*) to give ΔCt values. These values were then normalized to the ancestral parent values (ΔΔCt). Copy number was then determined as 2∧(−(ΔΔCt)). Values are the mean of three technical replicates with error bars showing standard deviation. “P” indicates CP1AB, “E” indicates evolved clone E1. The number (1–5 for P and 1–10 for E) indicates replicate evolved populations, and “a–c” indicate three randomly chosen end-point clones.
(TIF)

Figure S3 Specific Growth Rate of *HXT6/7* Segregants in Glucose-Rich Environments. Maximal specific growth rates of otherwise isogenic haploid segregants containing either wild type (4 segregants) or *HXT6/7* amplification (4 segregants) loci. The parent diploid of these strains was isogenic except for the *HXT6/7* amplification, based upon high-throughput sequencing.
(TIF)

Table S1 Competition Coefficients (Relative to Common Reference Strain) in Three Environments.
(XLS)

Table S2 Grand Means of Relative Fitness within Each Alternative Environment.
(XLS)

Table S3 Steady State Physiological Measurements within Each Alternative Environment.
(XLS)

Table S4 Summary of Illumina Sequence Data.
(XLS)

Table S5 Primers used.
(XLS)

Table S6 Significance Analysis of Mutations in Evolved Clones.
(XLS)

Table S7 Significance Analysis of Mutations in Coding Regions and Non-Synonymous Mutations.
(XLS)

Table S8 Competition Coefficients (Relative to Common Reference Strain) in Glucose-Rich Environments.
(XLS)
