# Supplementary figures and images for: Hunger Artists: Yeast Adapted to Carbon Limitation Show Trade-Offs under Carbon Sufficiency
Source: PLoS Genet. 2011 Aug 4;7(8):e1002202. doi: 10.1371/journal.pgen.1002202 (PMC3150441; doi:10.1371/journal.pgen.1002202)

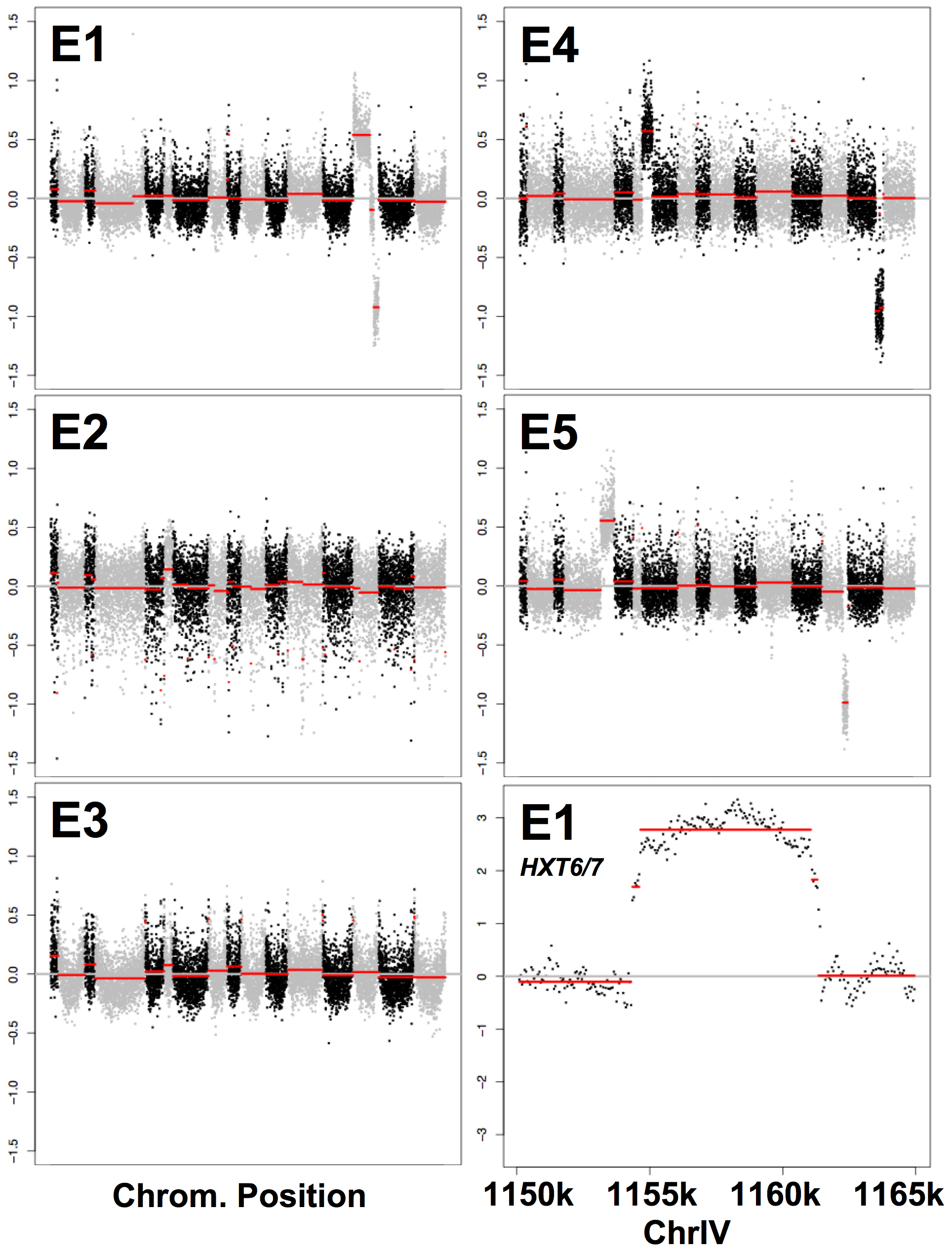

Supplement: Figure S1 — Evolved Copy Number Variations. Depth-of-coverage plots for E1 through E5, relative to the ancestral diploid CP1AB. Values plotted are log2 ratios of mean sequencing coverage in 1 kb windows across the genome (evolved/ancestral). Red lines represent segment means determined by DNAcopy (see Materials and Methods). (TIF) [file pgen.1002202.s003.tif]

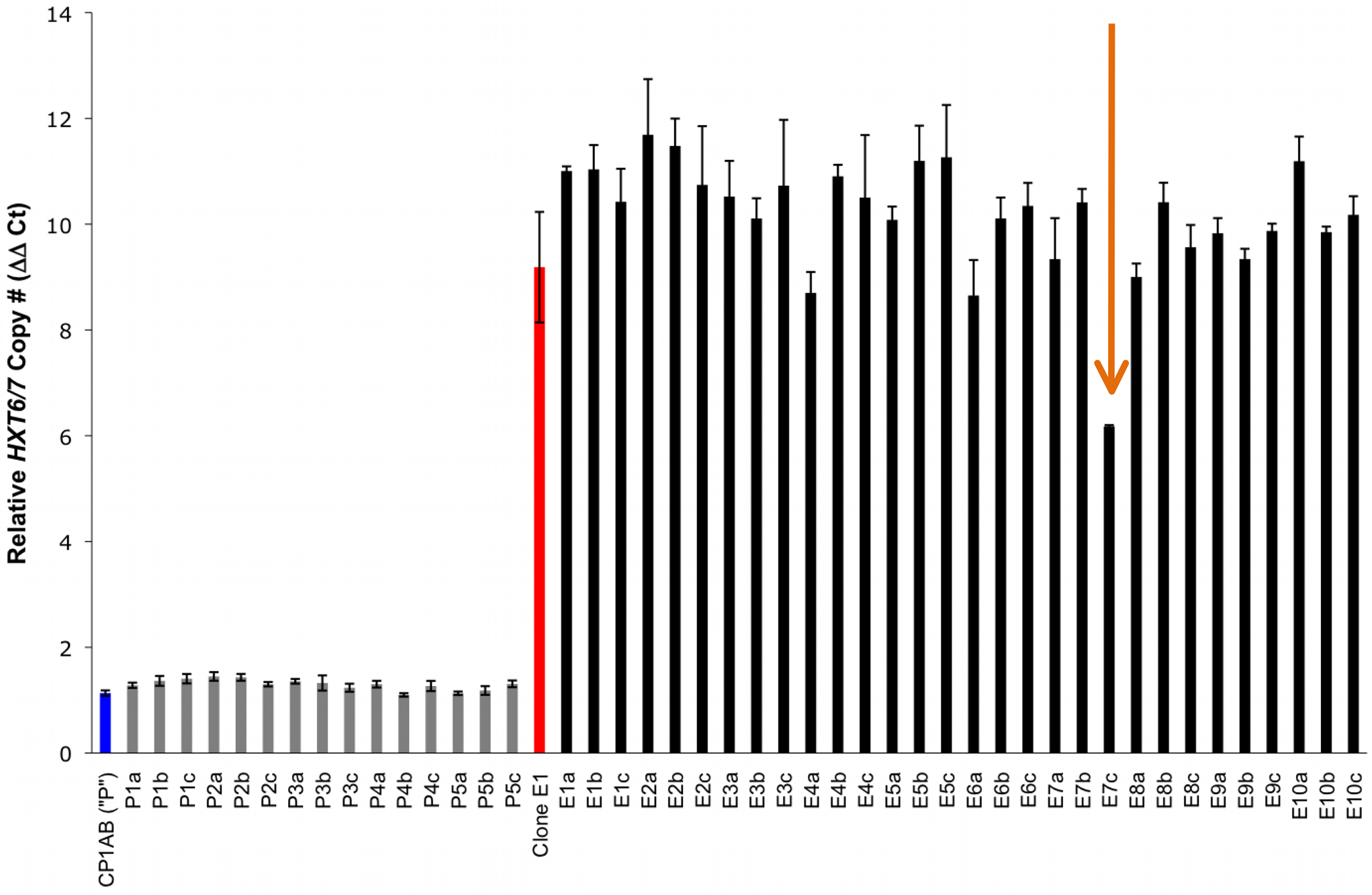

Supplement: Figure S2 — Copy Number of HXT6/7 locus following serial batch evolution under high glucose. Copy number of the HXT6/7 locus relative to the ancestral parent. Raw values for HXT6/7 locus were normalized to an internal control chrIV locus (UBP1) to give ΔCt values. These values were then normalized to the ancestral parent values (ΔΔCt). Copy number was then determined as 2∧(−(ΔΔCt)). Values are the mean of three technical replicates with error bars showing standard deviation. “P” indicates CP1AB, “E” indicates evolved clone E1. The number (1–5 for P and 1–10 for E) indicates replicate evolved populations, and “a–c” indicate three randomly chosen end-point clones. (TIF) [file pgen.1002202.s004.tif]

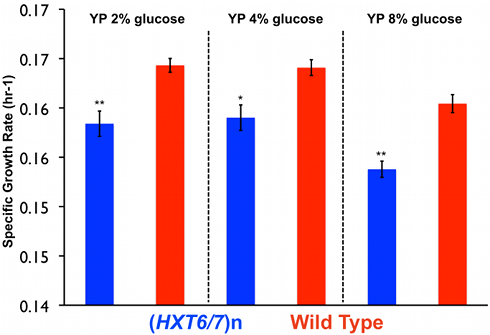

Supplement: Figure S3 — Specific Growth Rate of HXT6/7 Segregants in Glucose-Rich Environments. Maximal specific growth rates of otherwise isogenic haploid segregants containing either wild type (4 segregants) or HXT6/7 amplification (4 segregants) loci. The parent diploid of these strains was isogenic except for the HXT6/7 amplification, based upon high-throughput sequencing. (TIF) [file pgen.1002202.s005.tif]
